# Long-term outcomes after capitate fractures: a median 16-year follow-up

**DOI:** 10.1007/s00402-024-05495-z

**Published:** 2024-08-23

**Authors:** Daniel Ossowski, Niels O. B. Thomsen, Martin Clementson, Jack Besjakov, Peter Jörgsholm, Anders Björkman

**Affiliations:** 1 Department of Hand Surgery, Institute of Translational Medicine, Skåne University Hospital Malmö and Lund University, Jan Waldenströms gata 5, Malmö, 205 02 Sweden; 2 Mölholm Private Hospital, Brummersvej 1, Vejle, DK-7100 Danmark; 3 Department of Hand Surgery, Institute of Clinical Sciences, Sahlgrenska Academy, University of Gothenburg and Sahlgrenska University Hospital, Box 100, Göteborg, 405 30 Sweden

**Keywords:** Carpal bone fracture, Capitate, Scaphocapitate syndrome, Avascular necrosis, Wrist, Carpal bones

## Abstract

**Introduction:**

The long-term effects of a capitate fracture are unknown. The aim of this study was to assess both clinical and radiological long-term outcomes after a capitate fracture.

**Materials and methods:**

From a cohort of 526 consecutive patients with post traumatic radial sided wrist pain, 23 were identified diagnosed with a capitate fracture. 16 of these (11 males and 5 females) with a median age at injury of 17.5 years (range 11–27 years) were eligible for a follow-up after a median of 16 years (range 8 to 17 years). In this study patients were examined using conventional radiographs, computed tomography (CT) and magnetic resonance imaging (MRI) at the time of injury and with CT at the follow-up. At follow-up radiological signs of osteoarthritis were graded in four stages and clinical outcome was evaluated by measuring range of wrist motion and grip and pinch strength. The subjective outcome was assessed using DASH and PRWE questionnaires.

**Results:**

Five patients had isolated capitate fractures and 11 had concomitant fractures, 10 of which had a simultaneous scaphoid fracture. 14 patients had been treated non-surgically in a cast and two patients were treated surgically. None of the fractures were visible on conventional radiographs at the time of injury. At follow-up all fractures had healed without signs of avascular necrosis. In one patient, CT examination revealed osteoarthritis between the capitate and lunate. This did not cause clinical symptoms. Functional impairments and pain scores were low: median DASH score 0, median PRWE 3 and median VAS pain score 0. We found no impairment in range of motion or grip and pinch strength.

**Conclusions:**

At a median of 16-year follow-up, patients with a capitate fracture report normal self-assessed hand function as well as good wrist motion and strength. The risk of development of posttraumatic arthritis in the joints around the capitate is low.

## Introduction

The capitate is centered within the carpus and thus well protected from injury. Capitate fractures are rare, constituting approximately 8% of all carpal fractures [1]. The majority of these fractures are reported to occur in combination with other carpal fractures or with associated ligament injuries [2–6]. Capitate fractures can be difficult to diagnose with conventional radiographs because they are often non- or minimally displaced due to stabilization by intercarpal ligaments. Therefore, more advanced imaging, such as MRI or CT, are important [2, 7, 8]. The majority of epidemiological studies [9, 10] have used medical charts or conventional radiographs to describe carpal fracture prevalence, which therefore is likely to be underrated.

Four major fracture patterns have been described for the capitate. The most common type is a transverse fracture of the capitate body which is often seen in combination with a perilunate injury with or without a simultaneous scaphoid fracture [6, 11]. Other fracture patterns are: a transverse fracture of the proximal pole (waist), a coronal oblique fracture and finally a parasagittal fracture. Studies have suggested that the capitate is vulnerable to post-traumatic avascular necrosis because of its blood supply, as the vessels enter dorsally and supply the bone in retrograde fashion [12]. However, more recent studies, based on micro-CT angiography examination of the vascularity in cadaveric capitates, show that 70% of the capitates also have a vessel entering the proximal pole. This fact can explain why avascular necrosis (AVN) of the capitate is rare [13]. Nonetheless, undiagnosed and untreated, capitate fractures can lead to avascular necrosis [14, 15] and nonunion [3, 15, 16].

There are no prior studies where patients with capitate fractures have been randomized to conservative or surgical treatment. However, there is a general understanding that undislocated isolated fractures can be treated for at least 4 weeks in a cast, whereas an isolated dislocated fractures or capitate fractures combined with other carpal fractures benefit from surgical stabilization [2, 17, 18].

The most common complication after a capitate fracture is development of nonunion, which often is related to delay in diagnosis [16, 19–21]. In addition, the capitate is multiarticular except for its dorsal and palmar surfaces and a capitate fracture carries a risk for intraarticular cartilage damage and posttraumatic osteoarthritis [14, 22]. Knowledge concerning long-term clinical and radiological outcomes remains limited [3].

The aim of this study was to assess the long-term clinical outcomes following capitate fractures, and based on CT examinations, to evaluate malunion and development of osteoarthritis in the joints around the capitate.

## Method

### Patients

From 2004 to 2008, all patients presenting at the emergency ward at our hospital, with posttraumatic radial sided wrist pain following an injury, were asked to participate in the study [1]. The inclusion criterion was posttraumatic pain on the radial side of the wrist, located distal to the radiocarpal joint and proximal to the carpometacarpal. Exclusion criteria were: Injuries being a part of a radiocarpal or intercarpal fracture dislocation or intrinsic ligament disruption and injuries older than 14 days. All patients were examined according to a study specific protocol including conventional radiographs of the wrist. Regardless of the result from the radiographs all patients in the study underwent an MRI of the wrist within three working days after inclusion in the study. In addition, if the MRI revealed a scaphoid or a capitate fracture a computed tomography (CT) was performed. A total of 526 patients were examined [23]. From this cohort, 23 patients were identified having a capitate fracture. At the present long-term follow up two patients had died and one patient had moved abroad. Thus, 20 patients where accessible for clinical and radiological follow-up.

### Imaging

At inclusion, conventional radiographs of the wrist were taken in dorsovolar and lateral projections with an additional four views of the scaphoid. MRI was performed with a small joint coil using a 0.23 low Tesla field MRI unit (Proview Marconi Medical Systems, Vantaa Finland). The study protocol used coronal short tau inversion recovery (STIR) 3-mm slice thickness; coronal T1 field echo 3-dimensional (FE3D) 2 mm-slice thickness; axial T1 fast spin echo 3.5 mm slice thickness; sagittal T1 FE3D 2-mm slice thickness. A fracture was defined as a cortical and trabecular linear lesion, causing intramedullary hyperintensity on the STIR as well as intramedullary hypointensity on T1 weighted images, extending to the cortices. CT was performed using a 16-slice scanner (Somatotom Sensation 16; Siemens AG, Forchheim, Germany). The patient was positioned prone with the hand extended above the head (superman position). Axial sections of 0.6 mm thick slices were obtained from which 1–2 mm thick reconstructions in the coronal and sagittal planes were done.

At the present long-term follow-up, CT was performed with a 320-slice CT scanner (Aquilon ONE Genesis: Canon Medical Systems, Otawara, Japan) Patients were positioned prone in the superman position. Axial 0.5 mm thick sections were obtained. 2 mm thick reconstructions in the coronal and sagittal planes of the capitate were attained.

### Clinical evaluation and patient rated outcome measures (PROMs)

The clinical examination was done independently by specialist in hand surgery, author DO, who had not participated in the initial treatment of the patients. Also, the examination was done without knowledge of results from the present CT scan.

Patient rated outcome (PRO) were assessed with the Disability of Arm Shoulder and Hand (DASH) questionnaire [24] and the Patient Reported Wrist Evaluation (PRWE) form [25]. Pain was assessed using VAS score from the PRWE form.

Range of movement was measured using a goniometer. Grip strength was measured using an Exacta Hydraulic Hand Dynamometer (North Coast Medical, Gilroy, CA, USA). Pinch strength was measured with B&L pinch gauge (B & L Engineering, Tustin, CA, USA). The mean value of three measures was used for calculation.

Intracarpal instability was assessed with the Lichtman test [26], Watson shift test [27], and a ballottement test between capitate and lunate. All clinical evaluations were done on both hands.

### Radiological assessment

We used the classification system proposed by Kadar and co-workers where capitate fracture types are described depending on localization and pattern. Three main categories are described: capitate body fractures (further subdivided in stellate comminuted, oblique high and low, transverse high and low) avulsion tip fractures, and shear depression fractures [6].

**Fig. 1:**
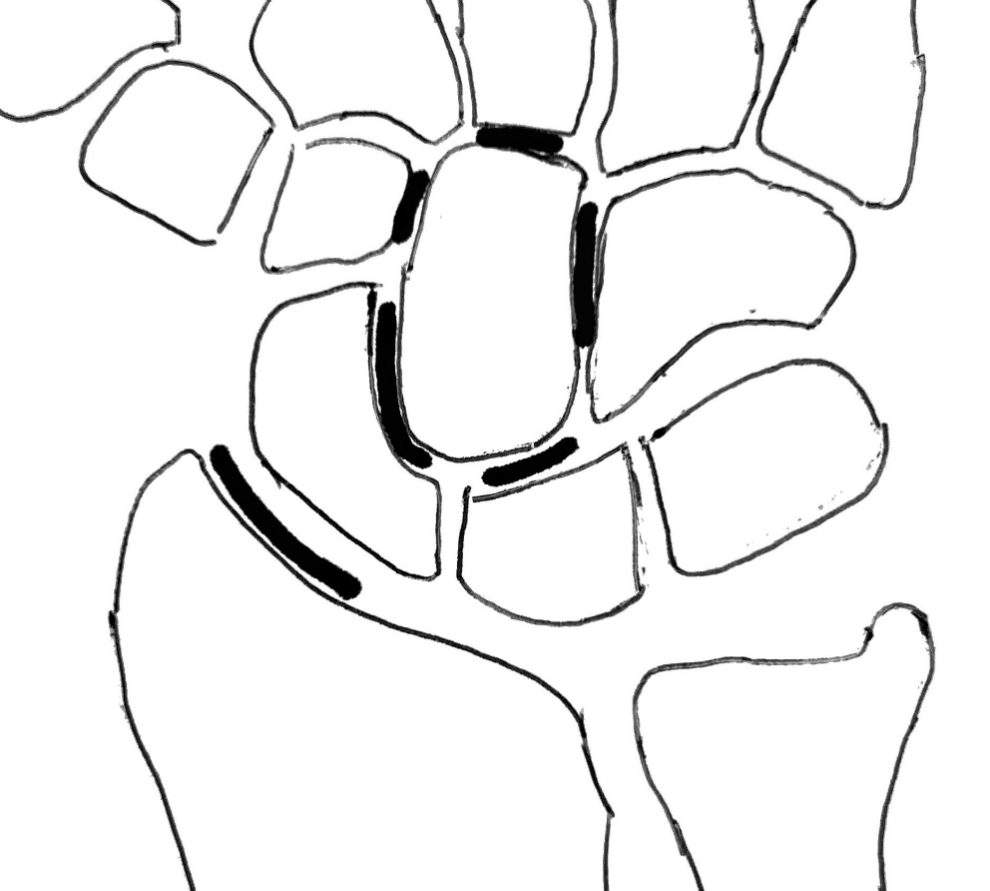
Articulations measured for osteoarthritis

From CT at follow-up, we evaluated fracture healing and malunion. The malunion was assessed in the capitate and in the scaphoid, the height–length ratio, lateral intrascaphoid angle, and dorsal cortical angle, were measured [28]. Signs of osteoarthritis were assessed in six defined articulations: capitate-scaphoid, scaphoid and radius, capitate and lunate, capitate and 3rd metacarpal, capitate and trapezoid, capitate and hamate (Fig. 1). Osteoarthritis was classified according to Whites classification as described by Clementson [29] (Table 1). Furthermore, the scapho-lunate (SL) interval and the SL angle were measured. All CT examinations were reviewed independently by two of the authors: JOB, senior specialist in musculoskeletal radiology and DO, senior specialist in hand surgery.

**Table 1:** Whites classification

Stage 0	Joint space < 50% reduction and no arthritic changes^a^
Stage 1	Joint space < 50% reduction + arthritic changes, or joint space > 50% reduction +/- subcortical sclerosis
Stage 2	Joint space > 50% reduction + arthritic changes
Stage 3	Joint attrition with bone-to-bone contact.

^a^ Subcortical sclerosis, osteophytes, or cysts

### Outcomes

The primary outcomes were DASH and PRWE. The secondary outcomes were: wrist range of motion, grip-strength, pinch strength, signs of osteoarthritis on CT examination and evaluation of fracture healing.

### Statistics

Data analysis was done using SPSS version 27 for Macintosh (IBM, Armonk, NY, USA). Statistical differences between medians were tested using Mann-Whitney U test. P-value less than 0.05 was considered statistically significant.

Interobserver agreement was calculated for arthritic staging according to Whites classification [30]. Interobserver agreement was analyzed with kappa statistics including calculations of CI. The resulting kappa value was 0.84, suggesting good interobserver agreement [31].

### Ethics

#### Ethical approval

was obtained from the Swedish Ethical Review Authority (ref. lu-459 03 and 2021 − 00957). The regional committee for radiation safety sanctioned the study. Informed written consent was obtained from all participants.

## Results

Of 20 patients asked to participate in this long-term follow-up, 3 declined and one patient could not be reached. Thus, the participants comprised 11 males and 5 females with a median (range) age of 17,5 years (11 to 27 years) at injury. Median (range) time from injury to follow up was 16 years (8 to 17 years) (Table 2).

**Table 2:** Demographics

Patient	Injured side	Gender	Fracture type	Trauma type	Age at injury (years)	Follow up (years)
1	Left	Female	Transverse low	Simple fall	11	18
2	Left	Female	Coronal shear	Simple fall	18	17
3	Right	Female	Transverse low	Simple fall	21	17
4	Right	Male	Transverse low	Simple fall	11	17
5	Right	Male	Oblique low	Simple fall	20	17
6	Left	Male	Transverse high	Bicycle	27	17
7	Left	Male	Oblique low	Skateboard	15	16
8	Right	Male	Transverse low	Simple fall	12	16
9	Left	Male	Transverse high	Soccer	15	16
10	Right	Female	Dorsal depression	Floor hockey	15	16
11	Left	Male	Transverse low	Bicycle	11	16
12	Right	Female	Oblique low	Fall in staircase	25	15
13	Left	Male	Oblique low	Ice hockey	18	15
14	Left	Male	Oblique low	Soccer	23	15
15	Right	Male	Oblique low	Skateboard	20	9
16	Right	Male	Transverse low	Basketball	17	14

The most common trauma mechanism was a fall to an outstretched extended hand in the same plane, as six patients fractured their capitate in a simple fall and seven patients acquired the injury in a fall during sport activity such as football, floorball, basketball, ice hockey or skateboard. Two patients had a bicycle injury and one patient acquired the fracture in a fall on a staircase.

Five patients had an isolated capitate fracture. Ten had a capitate fracture in combination with a scaphoid fracture, whereof nine were undislocated (dislocation below 0.5 mm) and one was dislocated. One patient had a capitate fracture in combination with fracture in the base of the 3rd and 4th metacarpals.

All fractures were diagnosed on MRI. None of the capitate fractures were visible on conventional radiographs but 4 out of 15 fractures were detected on the CT examination. One of the patients did not undergo initial CT examination. Of the 16 patients that were assessed at follow up all but two had been treated conservatively with a cast for 6 to 12 weeks. Two patients underwent surgery, one due to a concomitant dislocated scaphoid fracture and a Galeazzi-fracture, and one patient because of an associated TFCC injury.

### Clinical evaluation

There were no significant differences in wrist range of motion, or grip- and pinch strength between the injured an uninjured wrist (Table 3).

**Table 3:** Results at long-term follow up

Patient	DASH	PRWE	Grip strength, kg (% of uninjured side)	ROM Flexion-extension (% of uninjured side)	ROM ulnar-radial deviation (% of uninjured side)
1	1	5	35,5 (87%)	130° (100%)	70° (100%)
2	0	0	36 (100%)	135° (104%)	30° (150%)
3	0	12	30 (100%)	135° (96%)	40° (100%)
4	0	0	38 (100%)	140° (100%)	40° (100%)
5	2	16	56 (97%)	140° (100%)	70° (93%)
6^a^	13	21	32 (64%)	95° (76%)	35° (78%)
7	2	8	52 (118%)	130° (96%)	45° (113%)
8	0	0	70 (109%)	140° (104%)	45° (100%)
9	1	8	32 (76%)	150° (97%)	55° (85%)
10	2,5	9	22,5 (94%)	155° (100%)	75° (100%)
11^b^	2,5	1,5	38 (66%)	125° (96%)	45° (90%)
12^c^	33	29	30 (100%)	145° (97%)	45° (82%)
13	0	0	42 (124%)	145° (104%)	55° (122%)
14	0	0	46 (100%)	125° (104%)	55° (122%)
15	0	0	44 (85%)	165° (103%)	65° (118%)
16	0	0	46 (110%)	160° (97%)	55° (110%)

^a^Associated Galeazzi fracture

^b^At follow up signs of osteoarthritis and DISI

^c^Patient was diagnosed with a severe carpal tunnel syndrome prior to assessment in the study

One patient with an associated scaphoid fracture that healed with a slight humpback deformity, had signs of midcarpal instability with a positive Lichtman test.

### PROM

The median DASH score at follow up was 0 (range 0–33). The median PRWE was 3 (range 0–29). (Table 3) Two patients had DASH and PRWE scores indicating severe disability. One of these had carpal tunnel syndrome at the time of follow-up, while the other patient originally had more complex injury in terms of a capitate fracture in combination with a scaphoid- and Galeazzi-fracture.

### Radiology

At follow-up, and demonstrated by CT examination, all fractures were healed. One patient did not do a CT-examination at follow up due to pregnancy. No patient demonstrated radiological signs of avascular necrosis of the capitate.

**Fig. 2:**
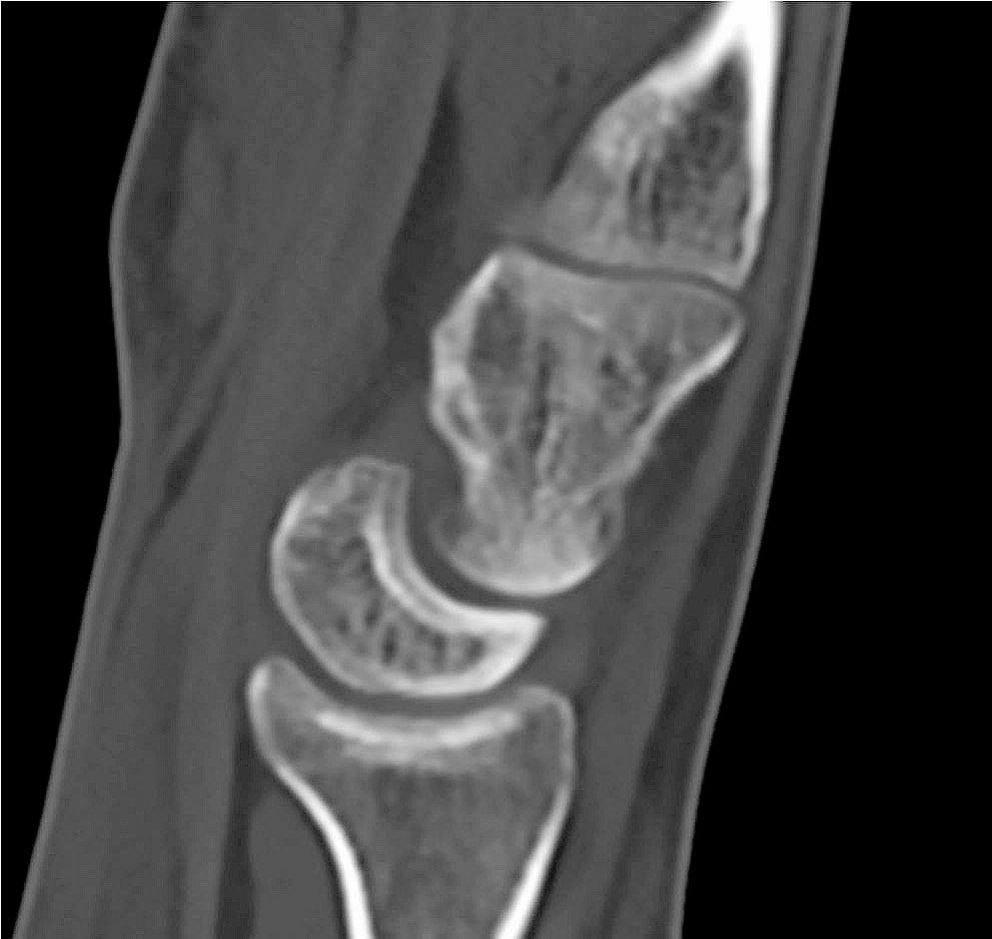
Patient no. 11 with signs of osteoarthritis and DISI

Only one patient demonstrated slight posttraumatic osteoarthritis which was located between the capitate and the lunate (Fig. 2). In this patient the capitate fracture had healed with a slight volar compression and the associated scaphoid fracture with humpback deformity. There was a scapholunate angle of 82 degrees. Clinically the patient had signs of midcarpal instability with a positive Lichtman test. The patient also had a moderate loss of grip strength (66% strength compared to the uninjured side) and pinch strength (64% compared to the uninjured side). Despite these findings, the patient rated hand function was excellent (DASH 2.5 and PRWE 1.5).

## Discussion

**Fig. 3:**
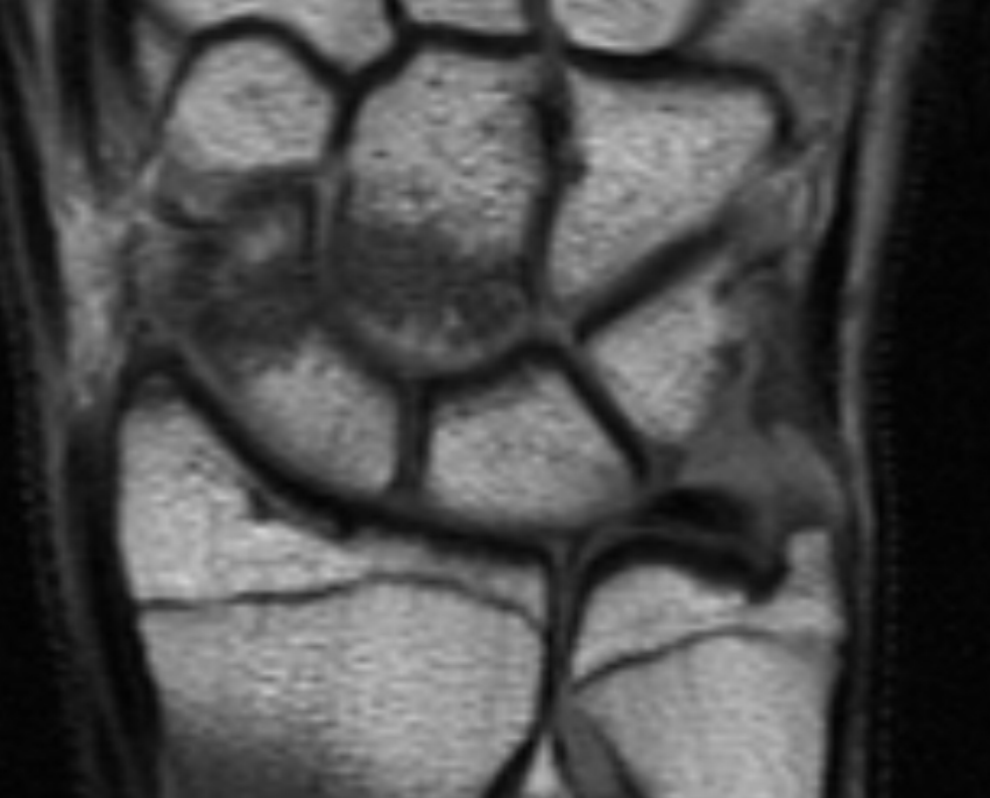
MR image of scaphoid and capitate fracture in patient no. 16

**Fig. 4:**
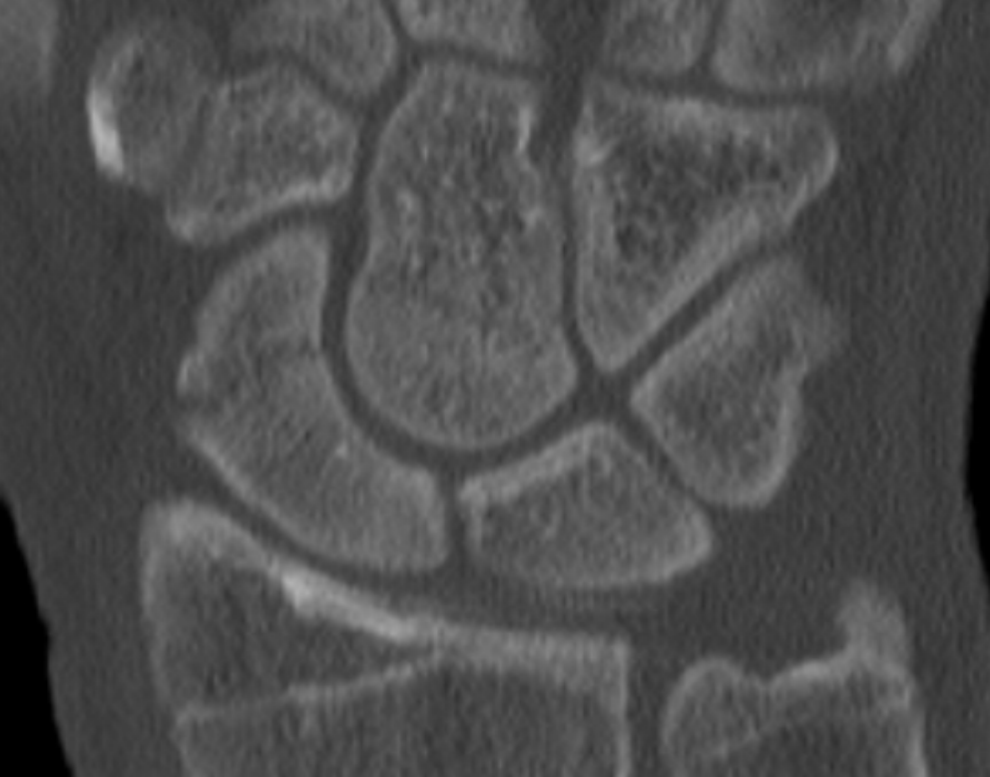
CT image of scaphoid and capitate fracture in patient no. 16

The majority of capitate fractures are seen in combination with a scaphoid fracture whereas isolated capitate fractures are reported to be rare [6, 32]. In this study 10 out of 16 patients had an associated scaphoid fracture (Figs. 3 and 4). Five patients out of 16 had an isolated capitate fracture which is a higher rate than reported in earlier studies. An explanation for the higher number of isolated fractures compared to prior literature could be that the diagnosis was based on MRI and not on conventional radiographs. Furthermore, fracture classification based on localization of the fracture might differ between imaging modalities. In our study, the diagnose and fracture classification are based on MRI. The oblique low (37.5%) and transverse low (37.5%) fracture were the most common fracture types. However, the small number of fractures in our study does not allow for statistical analyze of fracture types in relation to age or type of trauma.

The capitate is protected from trauma by the surrounding bones (and strong ligament attachments). However, this study shows that a capitate fracture alone or in combination with a scaphoid fracture, are most commonly caused by a simple fall in the same level, onto an extended wrist. One interesting question is why a fall to an outstretched hand result in a scaphoid and capitate fracture in a majority of cases whereas some only sustain an isolated capitate fracture. Prior biomechanical studies have suggested that depending on the position of the hand and wrist at the time of injury, the type and presentation of the capitate fracture varies [33]. Among patients with capitate and scaphoid fractures, Fenton [34] suggested that the scaphoid fracture occurs first and secondarily, if the injury force is not fully dissipated on the scaphoid, fracture of the neck of the capitate takes place. Based on biomechanical studies, were the wrist was forces in extension and ulnar deviation, Mayfield proposed a classification of perilunate dislocations and proposed that ligamentous injuries develop in a sequential fascion [35] Furthermore, Sterin and Siegel found in a cadaver specimen that fracture of the capitate could be produced in forced extension by the dorsal lip of the radius impacting on the capitate whereas the scaphoid fractured in tension created at the midcarpal joint level by forced extension [36]. Future biomechanical studies are needed for better understanding of the trauma mechanisms behind both isolated capitate fractures and capitate fractures combined with other injuries. However, it seems reasonable to assume that a combined scaphoid and capitate fracture indicates a more severe trauma requiring a closer follow-up in order to detect or exclude associated ligament injuries [37]. We found, one patient with radiologic osteoarthritis between the capitate and lunate (Fig. 2) The patient had a malunited scaphoid fracture, DISI configuration but no scapho-lunate dissociation. He also had a positive Lichtmans test. Given the patient status and what is known from biomechanical studies we believe that the injury is best described as a greater arc injury. However, this patient did not experience any subjective problems from the wrist.

Carpal fractures in children aged under 16 years are rare and among them, scaphoid fractures are most common [32, 38]. The median age of our patients with capitate fractures was 17.5 years which is younger than reported in a prior study [6] and also younger than reported for scaphoid fractures [39]. The capitate is the first carpal bone to start ossification which is completed at a mean age of 15.3 years in boys and 13.3 years in girls [40]. We speculate that the early ossification of the capitate is the reason for the low median age of fractures in our cohort.

It has been emphasized, that early diagnosis is important, as delayed treatment of capitate fractures can lead to avascular necrosis, nonunion, and post-traumatic osteoarthritis [3, 14]. Several studies advocate early MRI and/or CT examination in patients with post traumatic wrist pain but normal conventional radiographs [1, 41, 42]. Our study supports this recommendation since none of the capitate fractures were visible on conventional radiographs. However, only 4 of 16 capitate fractures were detected on CT. One explanation for the low detection rate on CT is that a 16-slice CT scanner was used. Based on our study, MRI is recommended when clinical suspicion of a capitate fracture exists, but CT results are normal. Newer MR scanners with higher signal-to-noise ratios are likely to enhance image quality. Additionally, dual-energy and photon-counting CT, now becoming standard, may significantly improve the diagnosis of capitate fractures. Future studies are needed to assess to role of MRI and CT in the diagnostic work-up of patients with suspected capitate fractures. Gelberman et al. [11] suggested that the capitate is vulnerable to post traumatic AVN due to its retrograde vascular supply. However, a recent study based on micro-CT angiography have shown than the capitate is also supplied by a more proximal vascular system. This suggests that individuals without a proximal vascular supply could have in increased risk for development of AVN [12, 14]. However, there are only few cases described with AVN after an isolated capitate fracture [2, 14] which is in accordance with our series where we did not find any case with AVN. All patients in our study were diagnosed and treated shortly of trauma. If left untreated, the most common fracture type in our study, the low transverse fracture, could gradually dislocate causing the head of the capitate to rotate with wrist movement. This could cause interruption of the vascular supply with the possibility to cause AVN or non-union [14, 15, 43, 44] Delayed union, malunion and non-union have been reported following capitate fractures [13]. Rand and colleagues found two non-unions in 11 fractures whereas Kadar and colleagues, only found one non-union out of 23 fractures corresponding to a 4% non-union rate [3, 6]. In addition, a literature review from 1999 described a total of 11 capitate nonunions [16]. Our long-term results support that early treated capitate fractures with no or minor dislocation carries low risk for developing pseudoarthrosis, malunion or avascular necrosis. In clinical practice nonunion of a prior non diagnosed capitate fracture is very rare. This supports the notion that minimally displaced capitate fractures, without other associated injuries, only need to be immobilized in a cast for the duration of pain. Our long-term results support that capitate fractures with no or minor dislocation, with or without a concomitant scaphoid fracture, treated with a cast at the time of injury carries low risk for developing pseudoarthrosis, malunion or avascular necrosis. In clinical practice nonunion of a prior non diagnosed capitate fracture is very rare. We speculate that minimally displaced capitate fractures without other injuries can be treated in a cast. A CT scan at 3–4 weeks can assess healing; if bridging callus is present, the fracture is stable and can be mobilized. However, capitate fractures with a concomitant scaphoid fracture may be more unstable and future studies are needed to assess if such injuries should be treated with longer cast immobilization.

Existing classifications systems for arthritis are solely based on conventional radiographs [30, 45] One previous study modified the White´s classification in order to include the additional information obtained from CT [29]. It allowed a more precise assessment of joint space narrowing as well as information on periarticular arthritic changes. Only one of our patients demonstrated signs of osteoarthrosis at the follow-up after a median of 16 years. This is in contrast to fractures of the scaphoid distal pole where osteoarthrosis in the STT (Scaphotrapeziotrapezoid) joint is more common [30]. It is well known that radiological signs of osteoarthritis not always leads to clinical symptoms. In accordance, our patient with radiological signs of arthritis between the capitate and lunate did not experience any clinical symptoms.

The strengths of our study are the inclusion of consecutive patients from a large prospective cohort and the use of conventional radiographs, CT and MR at diagnosis and CT for assessment at follow-up. However, some limitations are evident. Due to a focus on scaphoid fractures the inclusion criteria were posttraumatic radial sided carpal pain. Capitate fractures which present with central or ulnar pain may have been missed. Secondly, the number of capitate fractures is low, and it was not possible to recognize significant differences on outcome measures between fracture types. Finally, our modified classification system for osteoarthritis based on CT limits direct comparison with results from other studies.

## Conclusion

I this study, we found that patients with a capitate fracture were young, had acquired the fracture due to a fall in the same plane, and that the majority had a concomitant scaphoid fracture. Furthermore, the capitate fractures were often undislocated, and not visible on conventional radiographs. The risk of associated ligament injury and subsequent instability is low. The majority of capitate fractures can be treated in a cast and achieve excellent clinical result without risk of developing secondary arthritis or avascular necrosis.

## Data Availability

The data that support the findings of this study are not openly available due to reasons of sensitivity and are available from the corresponding author upon reasonable request. Data are located in controlled access data storage.

## References

[1] Jørgsholm P, Thomsen NO, Besjakov J, Abrahamsson SO, Björkman A (2013) The benefit of magnetic resonance imaging for patients with posttraumatic radial wrist tenderness. J Hand Surg Am 38(1):29–33. 10.1016/j.jhsa.2012.09.034 23200950 10.1016/j.jhsa.2012.09.034

[2] Papp S (2010) Carpal bone fractures. Hand Clin 26(1):119–127. 10.1016/j.hcl.2009.08.014 20006250 10.1016/j.hcl.2009.08.014

[3] Rand JA, Linscheid RL, Dobyns JH (1982) Capitate fractures: a long-term follow-up. Clin Orthop Relat Res (165):209–216 7075062

[4] Suh N, Ek ET, Wolfe SW (2014) Carpal fractures. J Hand Surg Am 39(4):785–791 quiz 791. doi. 10.1016/j.jhsa.2013.10.030 24679911 10.1016/j.jhsa.2013.10.030

[5] Apergis E, Darmanis S, Kastanis G, Papanikolaou A (2001) Does the term scaphocapitate syndrome need to be revised? A report of 6 cases. J Hand Surg Br 26(5):441–445. 10.1054/jhsb.2001.0589 11560426 10.1054/jhsb.2001.0589

[6] Kadar A, Morsy M, Sur YJ, Akdag O, Moran SL (2016) Capitate fractures: a review of 53 patients. J Hand Surg Am 41(10):e359–e366. 10.1016/j.jhsa.2016.07.099 27524693 10.1016/j.jhsa.2016.07.099

[7] Calandruccio JH, Duncan SF (1999) Isolated nondisplaced capitate waist fracture diagnosed by magnetic resonance imaging. J Hand Surg Am 24(4):856–859. 10.1053/jhsu.1999.0856 10447181 10.1053/jhsu.1999.0856

[8] Kussmaul AC, Kuehlein T, Langer MF, Ayache A, Löw S, Unglaub F The Conservative and Operative Treatment of Carpal Fractures. *Dtsch Arztebl Int*. Sep 2024;(Forthcoming):arztebl.m2024.0102. 10.3238/arztebl.m2024.0102 10.3238/arztebl.m2024.0102 PMC11661489 38863274

[9] Hove LM Fractures of the hand. Distribution and relative incidence (1993). Scand J Plast Reconstr Surg Hand Surg, 27(4):317–319 8159947

[10] Van Onselen EB, Karim RB, Hage JJ, Ritt MJ (2003) Prevalence and distribution of hand fractures. J Hand Surg Br 28(5):491–495. 10.1016/s0266-7681(03)00103-7 12954264 10.1016/s0266-7681(03)00103-7

[11] Dailiana ZH, Papatheodorou LK, Malizos KN (2015) Scaphocapitate fracture: two cases with Follow-Up over 5 years. J Wrist Surg 4(3):174–178. 10.1055/s-0035-1549290 26261742 10.1055/s-0035-1549290 PMC4530178

[12] Gelberman RH, Gross MS (1986) The vascularity of the wrist. Identification of arterial patterns at risk. Clin Orthop Relat Res, (202):40–49 3514029

[13] Kadar A, Morsy M, Sur YJ, Laungani AT, Akdag O, Moran S (2017) The vascular anatomy of the Capitate: New discoveries using Micro-computed Tomography Imaging. J Hand Surg Am 42(2):78–86. 10.1016/j.jhsa.2016.12.002 28160904 10.1016/j.jhsa.2016.12.002

[14] Vander Grend R, Dell PC, Glowczewskie F, Leslie B, Ruby LK (1984) Intraosseous blood supply of the capitate and its correlation with aseptic necrosis. J Hand Surg Am 9(5):677–683. 10.1016/s0363-5023(84)80012-x 6386955 10.1016/s0363-5023(84)80012-x

[15] Kadar A, Iordache SD (2021) Neglected Scaphocapitate Syndrome. J Wrist Surg 12(02):143–146. 10.1055/s-0041-1740402 36923103 10.1055/s-0041-1740402 PMC10010893

[16] Rico AA, Holguin PH, Martin JG (1999) Pseudarthrosis of the capitate. J Hand Surg Br 24(3):382–384. 10.1054/jhsb.1998.0056 10433464 10.1054/jhsb.1998.0056

[17] Minami M, Yamazaki J, Chisaka N, Kato S, Ogino T, Minami A (1987) Nonunion of the capitate. J Hand Surg Am 12(6):1089–1091. 10.1016/s0363-5023(87)80120-x 3320174 10.1016/s0363-5023(87)80120-x

[18] Richter B (2018) Fractures of the Hand and Carpus. 2018:269–271

[19] Reigstad O, Grimsgaard C, Thorkildsen R, Reigstad A, Røkkum M (2012) Scaphoid non-unions, where do they come from? The epidemiology and initial presentation of 270 scaphoid non-unions. Hand Surg 17(3):331–335. 10.1142/s0218810412500268 23061941 10.1142/S0218810412500268

[20] Freeman BH, Hay EL (1985) Nonunion of the capitate: a case report. J Hand Surg Am 10(2):187–190. 10.1016/s0363-5023(85)80102-7 3884694 10.1016/s0363-5023(85)80102-7

[21] De Schrijver F, De Smet L (2002) Isolated fracture of the capitate: the value of MRI in diagnosis and follow up. Acta Orthop Belg 68(3):310–315 12152383

[22] Vigler M, Aviles A, Lee SK (2006) Carpal fractures excluding the scaphoid. Hand Clin 22(4):501–516 abstract vii. doi. 10.1016/j.hcl.2006.07.007 17097470 10.1016/j.hcl.2006.07.007

[23] Jørgsholm P, Thomsen NO, Besjakov J, Abrahamsson SO, Björkman A (2013) The benefit of magnetic resonance imaging for patients with posttraumatic radial wrist tenderness. J Hand Surg Am 38(1):29–33. 10.1016/j.jhsa.2012.09.034 23200950 10.1016/j.jhsa.2012.09.034

[24] Hudak PL, Amadio PC, Bombardier C (1996) Development of an upper extremity outcome measure: the DASH (disabilities of the arm, shoulder and hand) [corrected]. The Upper Extremity Collaborative Group (UECG). Am J Ind Med 29(6):602–608. 10.1002/(sici)1097-0274(199606)29:6%3C602::Aid-ajim4%3E3.0.Co;2-l 8773720 10.1002/(SICI)1097-0274(199606)29:6<602::AID-AJIM4>3.0.CO;2-L

[25] MacDermid JC, Turgeon T, Richards RS, Beadle M, Roth JH (1998) Patient rating of wrist pain and disability: a reliable and valid measurement tool. J Orthop Traum 12(8):577–586. 10.1097/00005131-199811000-00009 10.1097/00005131-199811000-00009 9840793

[26] Feinstein WK, Lichtman DM, Noble PC, Alexander JW, Hipp JA (1999) Quantitative assessment of the midcarpal shift test. J Hand Surg Am 24(5):977–983. 10.1053/jhsu.1999.0977 10509276 10.1053/jhsu.1999.0977

[27] Watson HK, Ashmead Dt, Makhlouf MV (1988) Examination of the scaphoid. J Hand Surg Am 13(5):657–660. 10.1016/s0363-5023(88)80118-7 3241033 10.1016/s0363-5023(88)80118-7

[28] Bain GI, Bennett JD, MacDermid JC, Slethaug GP, Richards RS, Roth JH (1998) Measurement of the scaphoid humpback deformity using longitudinal computed tomography: intra- and interobserver variability using various measurement techniques. J Hand Surg Am 23(1):76–81. 10.1016/s0363-5023(98)80093-2 9523959 10.1016/S0363-5023(98)80093-2

[29] Clementson M, Thomsen N, Besjakov J, Jørgsholm P, Björkman A (2017) Long-term outcomes after distal scaphoid fractures: a 10-Year Follow-Up. J Hand Surg Am 42(11):927e. 10.1016/j.jhsa.2017.06.016 10.1016/j.jhsa.2017.06.016 28733100

[30] White L, Clavijo J, Gilula LA, Wollstein R (2010) Classification system for isolated arthritis of the scaphotrapeziotrapezoidal joint. Scand J Plast Reconstr Surg Hand Surg 44(2):112–117. 10.3109/02844311003675388 20465511 10.3109/02844311003675388

[31] Landis JR, Koch GG (1977) The measurement of observer agreement for categorical data. Biometrics 33(1):159–174 843571

[32] Weber DM, Kraus R, Wirth-Welle R et al (2023) Paediatric fractures of carpal bones other than the scaphoid. Hand Surg Rehabilitation. 10.1016/j.hansur.2023.06.009 10.1016/j.hansur.2023.06.009 37356568

[33] Yoshihara M, Sakai A, Toba N, Okimoto N, Shimokobe T, Nakamura T (2002) Nonunion of the isolated capitate waist fracture. J Orthop Sci 7(5):578–580. 10.1007/s007760200103 12355134 10.1007/s007760200103

[34] Fenton RL (1956) The naviculo-capitate fracture syndrome. J Bone Joint Surg Am, 38–a(3):681–4 13319423

[35] Mayfield JK, Johnson RP, Kilcoyne RK (1980) Carpal dislocations: pathomechanics and progressive perilunar instability. J Hand Surg Am 5(3):226–241. 10.1016/s0363-5023(80)80007-4 7400560 10.1016/s0363-5023(80)80007-4

[36] Stein F, Siegel MW (1969) Naviculocapitate fracture syndrome. A case report: new thoughts on the mechansim of injury. J Bone Joint Surg Am 51(2):391–395 5767329

[37] Jørgsholm P, Thomsen NO, Björkman A, Besjakov J, Abrahamsson SO (2010) The incidence of intrinsic and extrinsic ligament injuries in scaphoid waist fractures. J Hand Surg Am 35(3):368–374. 10.1016/j.jhsa.2009.12.023 20193857 10.1016/j.jhsa.2009.12.023

[38] Brudvik C, Hove L (2003) Childhood fractures in Bergen, Norway: identifying high-risk groups and activities. J Pediatr Orthop 23:629–634. 10.1097/00004694-200309000-00010 12960626 10.1097/00004694-200309000-00010

[39] Jørgsholm P, Ossowski D, Thomsen N, Björkman A (2020) Epidemiology of scaphoid fractures and non-unions: a systematic review. Handchir Mikrochir Plast Chir 52(5):374–381. 10.1055/a-1250-8190 32992390 10.1055/a-1250-8190

[40] Stuart HC, Pyle SI, Cornoni J, Reed RB (1962) Onsets, completions and spans of ossification in the 29 bonegrowth centers of the hand and wrist. Pediatrics 29:237–249 13917935

[41] Bergh TH, Lindau T, Bernardshaw SV et al (2012) A new definition of wrist sprain necessary after findings in a prospective MRI study. Injury 43(10):1732–1742. 10.1016/j.injury.2012.06.028 22819266 10.1016/j.injury.2012.06.028

[42] Courcey Cd, Jester A, Kaur S, Lindau TR, Oestreich K (2022) Early MRI for Pediatric wrist injuries—prospective Case Series of 150 cases. J Wrist Surg 12(02):096–103. 10.1055/s-0042-1753508 10.1055/s-0042-1753508 PMC10010895 36926210

[43] Bekele W, Escobedo E, Allen R (2011) Avascular necrosis of the capitate. J Radiol Case Rep 5(6):31–36. 10.3941/jrcr.v5i6.760 22470799 10.3941/jrcr.v5i6.760 PMC3303341

[44] Kaulesar Sukul DM, Johannes EJ (1992) Transscapho-transcapitate fracture dislocation of the carpus. J Hand Surg Am 17(2):348–353. 10.1016/0363-5023(92)90418-o 1564286 10.1016/0363-5023(92)90418-o

[45] Crosby EB, Linscheid RL, Dobyns JH (1978) Scaphotrapezial trapezoidal arthrosis. J Hand Surg Am 3(3):223–234. 10.1016/s0363-5023(78)80086-0 659818 10.1016/s0363-5023(78)80086-0

